# Phytosulfokine contributes to suspension culture of *Cunninghamia lanceolata* through its impact on redox homeostasis

**DOI:** 10.1186/s12870-023-04496-1

**Published:** 2023-10-09

**Authors:** Zhaodong Hao, Jinyu Shi, Hua Wu, Yiqing Yan, Kaifei Xing, Renhua Zheng, Jisen Shi, Jinhui Chen

**Affiliations:** 1 State Key Laboratory of Tree Genetics and Breeding, Co-Innovation Center for Sustainable Forestry in Southern China, Nanjing Forestry University, Nanjing, 210037 China; 2 Key Laboratory of Forest Genetics and Biotechnology of Ministry of Education, Nanjing Forestry University, Nanjing, 210037 China; 3 Fujian Academy of Forestry, Fuzhou, 350012 Fujian China

**Keywords:** *C. lanceolate*, PSK, Suspension culture, ROS, Bowning

## Abstract

**Background:**

Suspension culture is widely used in the establishment of efficient plant regeneration systems, as well as in the mass production of plant secondary metabolites. However, the establishment of a suspension culture system of *Cunninghamia lanceolata* is genotype-dependent given that proembryogenic masses (PEMs) are prone to browning during this process in recalcitrant genotypes. Previously, we reported that the plant peptide hormone phytosulfokine (PSK) can tremendously decrease the hydrogen peroxide (H_2_O_2_) level and help to initiate somatic embryogenesis (SE) in recalcitrant *C. lanceolata* genotypes. However, to date, no studies have revealed whether or how PSK may contribute to the establishment of a suspension culture system in these recalcitrant genotypes.

**Results:**

Here, we demonstrated that exogenous application of PSK effectively inhibited PEM browning during suspension culture in a recalcitrant genotype of *C. lanceolata*. Comparative time-series transcriptome profiling showed that redox homeostasis underwent drastic fluctuations when PEMs were cultured in liquid medium, while additional PSK treatment helped to maintain a relatively stable redox homeostasis. Interestingly, PSK seemed to have a dual effect on peroxidases (PRXs), with PSK simultaneously transcriptionally repressing ROS-producing PRXs and activating ROS-scavenging PRXs. Furthermore, determination of H_2_O_2_ and MDA content, as well as cell viability, showed that exogenous PSK treatment inhibited PEM browning and safeguarded PEM suspension culture by decreasing the H_2_O_2_ level and increasing PEM activity.

**Conclusions:**

Collectively, these findings provide a valuable tool for the future establishment of large-scale *C. lanceolata* PEM suspension culture without genotype limitations.

**Supplementary Information:**

The online version contains supplementary material available at 10.1186/s12870-023-04496-1.

## Background

Somatic embryogenesis (SE) is a unique process in plants in which embryos form from somatic cells and develop into whole plants in a developmental path that closely resembles zygotic embryogenesis both morphologically and temporally [1]. Since its first report in research on carrot in 1958, SE has been believed to be one of the most powerful biotechnology tools and is widely used in both basic research and applied research [2]. One SE application is in the production of plants, especially in crops and forest trees, for commercial-scale clonal propagation [3]. SE can be classified into two general modes, i.e., direct and indirect SE, depending on whether or not embryogenic calli (or proembryogenic masses, PEMs) are formed [4]. In addition, a sharp distinction between these two SE pathways is that the propagation efficiency is much higher in indirect SE than in direct SE, which is important to consider when laying the foundation for application in industrialized seedling cultivation [5, 6]. However, embryogenic calli are normally induced to form somatic embryos and develop into whole regenerated plants on a solid medium during indirect SE, which cannot be used for large-scale propagation due to the insufficient regeneration efficiency and the poor synchrony of somatic embryo development [6]. An alternative method is to culture and propagate embryogenic calli in liquid medium, namely, with an embryogenic cell suspension culture system that has been well established for mass proliferation in many plant species, such as *Euonymus alatus*, coconut, and date palm [7–9]. In addition, embryogenic cell suspension culture together with successive plant regeneration systems via SE has provided an ideal platform for genetic transformation in multiple species, such as in citrus, *Coffea arabica*, and *Liridoendron* [10–12].

*Cunninghamia lanceolata*, also known as Chinese fir, is one of the main plantation trees in southern China, with an artificial forest area of 148 million acres and a forest stock volume of 755 million m^3^ according to the 9th National Inventory of Forest Resources (2014–2018) in China. The SE system for *C. lanceolata* was established in 2017, in which PEMs that had been induced from immature cones were in turn induced to form somatic embryos on solid medium supplemented with polyethylene glycol (PEG) and abscisic acid (ABA) [13, 14]. Furthermore, *C. lanceolata* PEMs have been synchronized in liquid suspension before somatic embryo induction, leading to the improvement of successive SE both in quantity and quality [14]. However, not all genotypes are suitable for suspension culture since PEMs of recalcitrant genotypes are prone to browning, which causes loss of cell viability and ultimately cell death. Browning is a ubiquitous phenomenon in plant cell culture, representing a primary barrier in the establishment of suspension culture systems [15, 16]. Browning in plant tissues is usually induced by cell/tissue damage that leads to the enzymatic oxidation of soluble phenolic compounds into quinones by polyphenol oxidases (PPOs) [17]. Normally, antioxidants and adsorbents are added to the medium to decrease the degree of browning [15, 18]. However, these additives sometimes have deleterious effects on cell cultures and have to be removed from the culture medium at a certain point [19, 20].

Phytosulfokine (PSK) belongs to a group of plant peptides and has been classified as a growth factor with extensive functions in growth regulation, stress responses, and reproductive development [21]. PSK was first identified as a chemical compound that was secreted into the older cell suspensions and that enabled the feeder effect to be overcome in suspension-cultured plant cells [22, 23]. In higher plants, *PSK* genes encode PSK precursors that are sulphated in the cis-Golgi and proteolytically processed in the apoplast [24, 25]. Mature PSK peptides are recognized by PSK receptors (PSKRs), which belong to the leucine-rich repeat receptor-like protein kinase (LRR-RLK) family [26]. In tomato, PSKR1 perceives PSK and increases cytosolic [Ca^2+^], leading to auxin-mediated immune responses [27]. Further research showed that PSKR1 interacts with the calcium-dependent protein kinase CPK28, leading to phosphorylation of two sites in glutamine synthetase GS2, one of which regulates plant defence and the other of which regulates growth in tomato [28]. In addition, in response to chilling stress, PSK application decreases reactive oxygen species (ROS) content in peach and increases proline and γ-aminobutyric acid (GABA) contents in banana [29, 30]. In our previous study, we found that PSK inhibits PEM browning and decreases H_2_O_2_ content, leading to the improvement of the SE system, especially in recalcitrant *C. lanceolata* genotypes. Here, by combining exogenous application of PSK, phenotype characterization, histological staining and comparative transcriptomics, we explored the potential function of PSK in the establishment of PEM suspension culture in a recalcitrant *C. lanceolata* genotype to investigate the maintenance of redox homeostasis and prevention of PEM browning.

## Results

### *C. lanceolata* PEM suspension culture and sampling

**Fig. 1:**
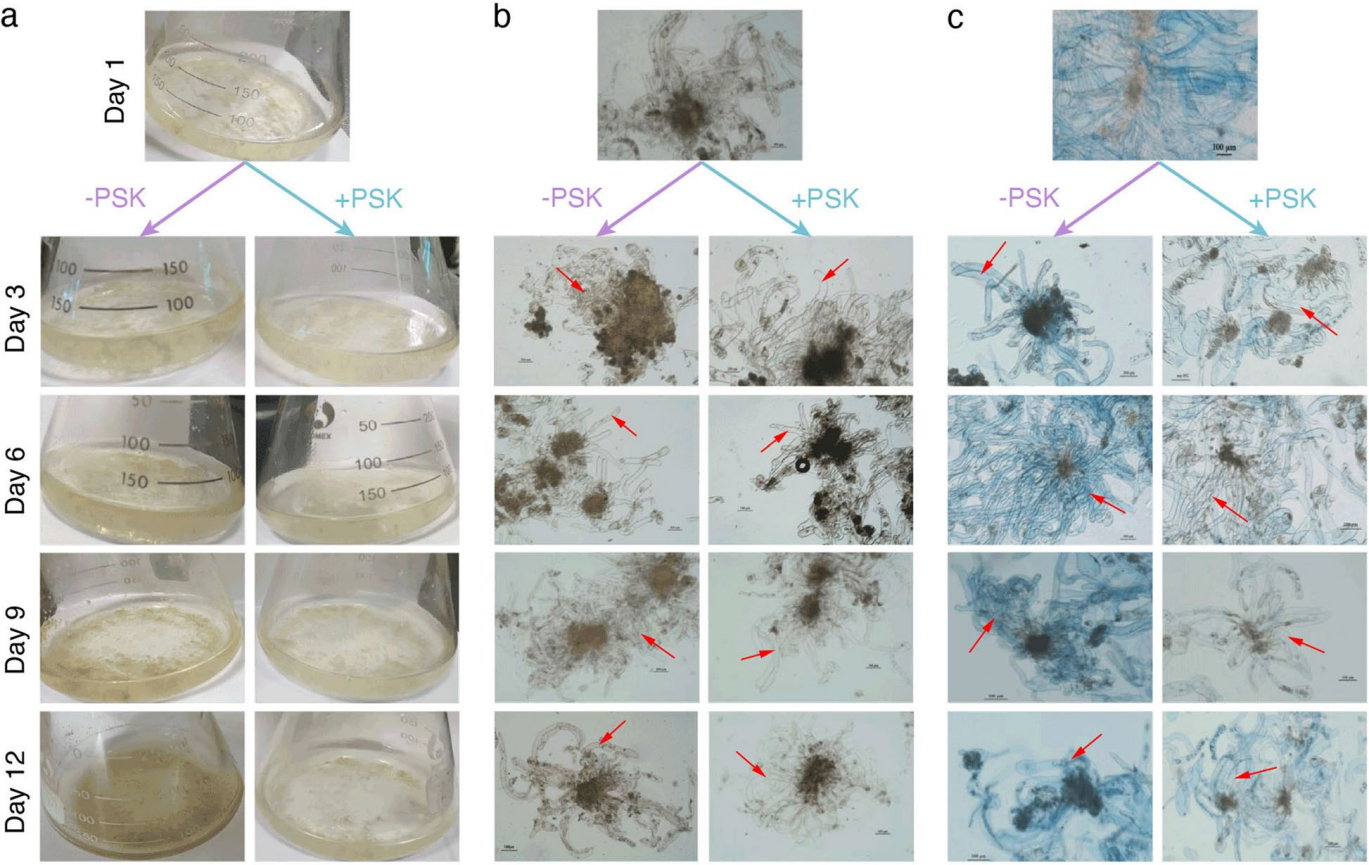
PSK inhibits PEM browning during suspension culture in the recalcitrant genotype of *C. lanceolata*. *C. lanceolata* PEMs of the recalcitrant genotype 4098–5 were cultured in liquid medium with or without exogenous PSK **a** The H_2_O_2_ accumulation and cell viability of PEM cultures were examined via DAB staining **b** and Evans blue staining **c**

*C. lanceolata* PEMs consist of a clump of rounded densely cytoplasmic cells with several highly vacuolated cells [31]. In general, PEMs are transferred to the solid SE induction medium and induced to form somatic embryos [14]. However, this solid induction method is not suitable for large-scale production because of its limited reproductive efficiency and poor synchronization. Thus, it is important to develop a liquid suspension culture system with characteristics of rapid proliferation. Here, we established a PEM suspension culture system using the recalcitrant *C. lanceolata* genotype 4098–5, which is prone to browning when cultured in liquid medium but remains normal with additional exogenous PSK (Fig. 1a). 3,3'-Diaminobenzidine (DAB) staining showed obviously decreased H_2_O_2_ levels in the PEM cultures receiving PSK treatment compared to the nontreatment control (Fig. 1b). In addition, Evans blue staining indicated that the PEMs were less active under control conditions than under PSK treatment (Fig. 1c). These results revealed that PSK helped to inhibit ROS burst and to promote cell viability when the PEMs were cultured in liquid medium. To investigate the potential role of PSK in this process, we collected PEM cultures at 1, 3, 6, 9, and 12 days in liquid medium with or without PSK treatment for RNA sequencing (RNA-seq).

### Comparative time-series transcriptome profiling

To examine the potential role of PSK in *C. lanceolata* PEM suspension cultures, we generated two sets of time-series RNA-seq data from 1 to 12 days with or without PSK treatment. A total of 181.6 Gb clean data was obtained with RNA-seq data of each sample ranging from 5.8 to 7.4 Gb (Table S1). By de novo transcript assembly, we obtained a total of 208,714 transcripts and 99,295 unigenes with average lengths of 1,197 bp and 957 bp, respectively (Table S2). Assembly completeness assessment using Benchmarking Universal Single-Copy Orthologs (BUSCO) showed that complete BUSCOs accounted for 84.44% and 70.97%, respectively (Table S3).

**Fig. 2:**
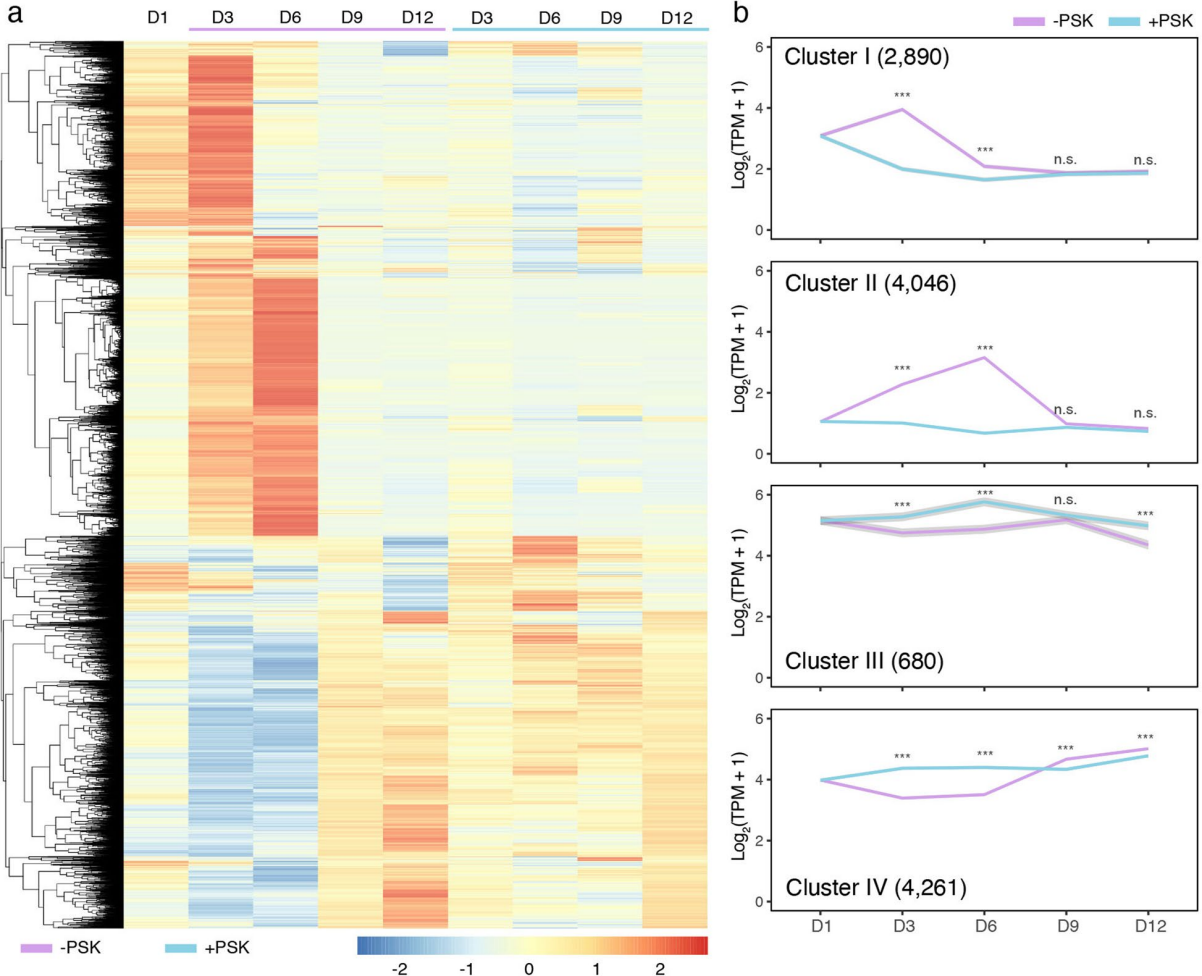
Transcriptional comparison of *C. lanceolata* PEM cultures between the PSK treatment and nontreatment control. Differentially expressed unigenes were hierarchically clustered using the expression values **a** K-means clustering analysis classified these differentially expressed unigenes into four clusters according to their expression profiles under control conditions **b** A paired t test was performed at each time point between the PSK treatment and nontreatment control

Among these 99,295 unigenes, 43.79% (43, 481 unigenes) were expressed in at least one stage across all time points in the PSK treatment and nontreatment groups. We identified differentially expressed unigenes via one-way analysis of variance (ANOVA) tests with a false discovery rate [FDR] less than 0.05, leading to 11,877 unigenes. The hierarchical clustering of these differentially expressed unigenes showed that PEMs underwent extensive transcriptional changes in the liquid medium without PSK, while most of these unigenes maintained a stable expression pattern in the PSK treatment (Fig. 2a). Then, we used the K-means clustering algorithm to obtain four clusters based on the gene expression profiles under control conditions (Fig. 2b). For each cluster, we extracted and examined gene expression patterns in the PSK treatment and performed a pairwise comparison between the PSK treatment and nontreatment control at each time point. Consistent with the hierarchical clustering results, the pairwise comparison showed that most unigenes changed dramatically at the transcriptional level in the nontreatment control but remained relatively stable in the PSK treatment. In particular, 24% (2,890, Cluster I) and 34% (4,046, Cluster II) of unigenes were first significantly upregulated and then significantly downregulated and peaked at Days 3 and 6, respectively, under control conditions. In contrast, expression levels of unigenes in these two clusters were slightly decreased and remained stable in general in the PSK treatment.

### Redox fluctuation during PEM suspension culture

**Fig. 3:**
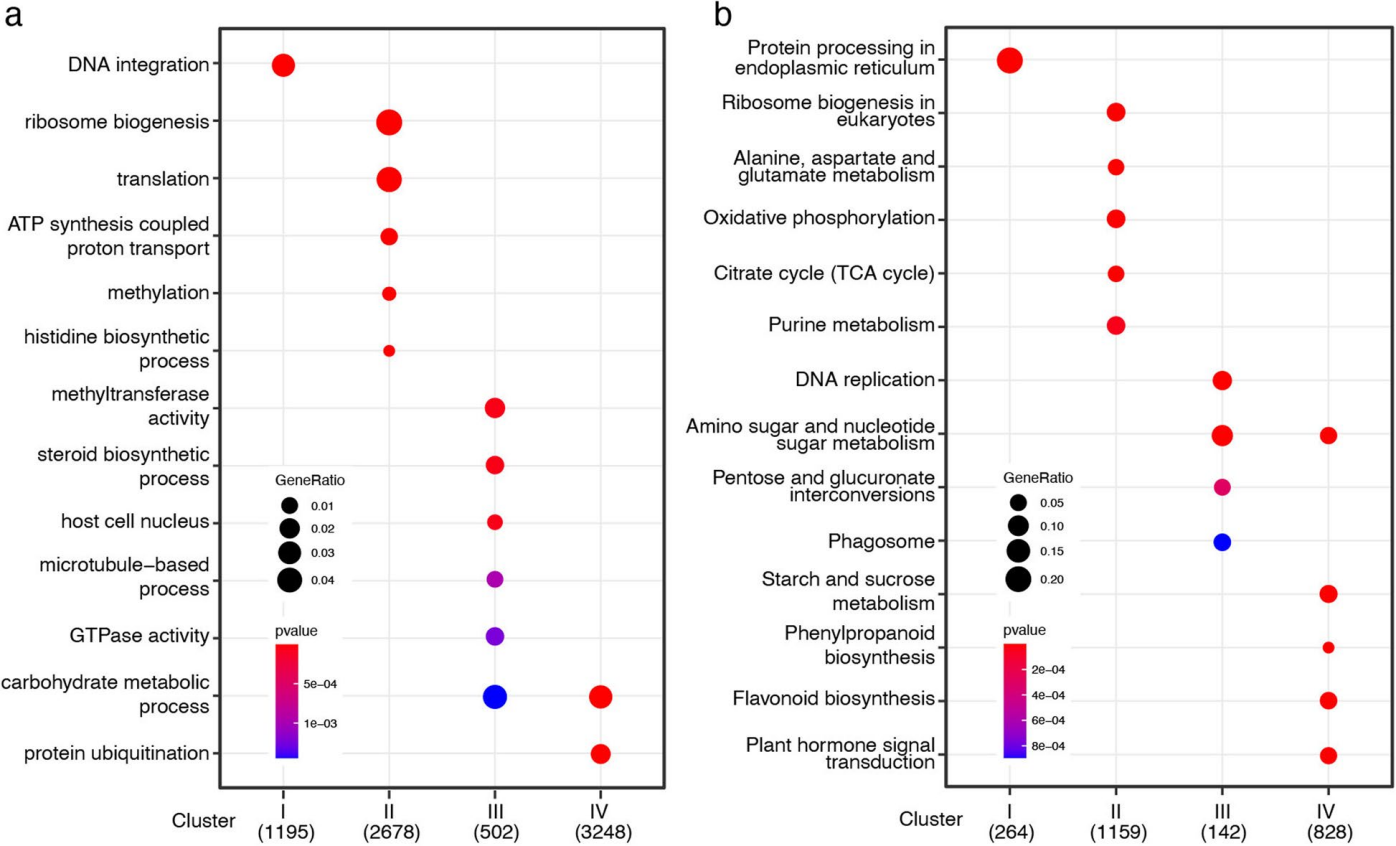
Comparison of the functional enrichment of four clusters with distinct expression patterns. GO **a** and KEGG **b** functional enrichment analyses were performed and compared across four unigene clusters with distinct expression patterns

To further explore the potential biological mechanisms underlying these clusters, we performed comparative functional enrichment analysis (Fig. 3). Both Gene Ontology (GO) [32] and Kyoto Encyclopedia of Genes and Genomes (KEGG) [33] enrichment analyses showed that Clusters I and II were enriched in cell growth- and proliferation-related terms such as ribosome biogenesis (GO:0042254, ko03008) and protein processing in the endoplasmic reticulum (ko04141). Meanwhile, Cluster II was also enriched in energy metabolism-related terms, such as ATP synthesis coupled proton transport (GO:0015986), the TCA cycle (ko00020) and oxidative phosphorylation (ko00190). These results indicated that PEMs cultured in liquid medium led to enhanced cellular respiration under control conditions but not under the PSK treatment.

**Fig. 4:**
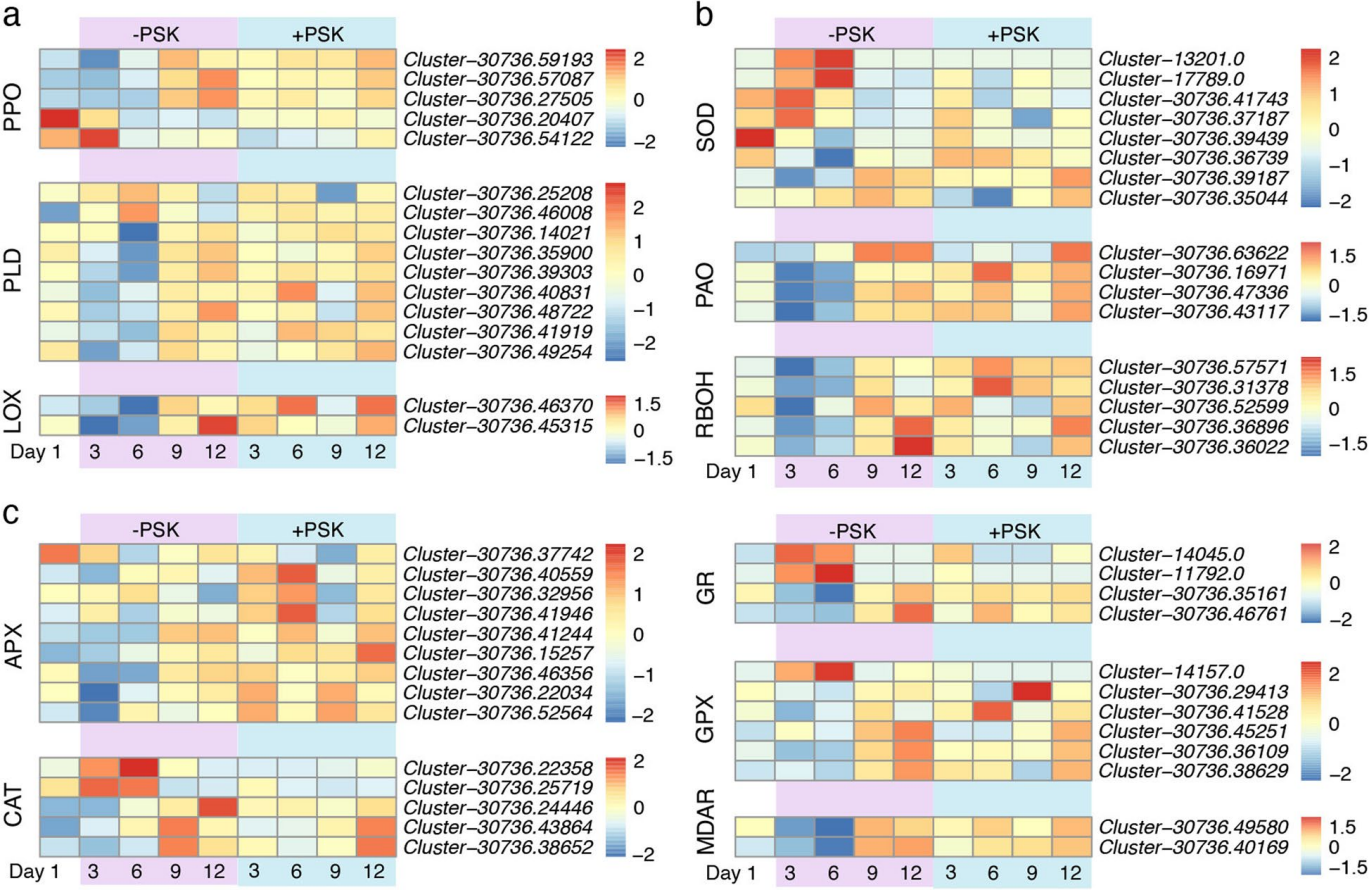
Transcriptional comparisons of redox homeostasis-related enzymes. The expression heatmap for the main enzymes that participate in enzymatic browning **a** ROS production **b** and ROS scavenging **c** PPO, polyphenol oxidase; PLD, phospholipase D; LOX, lipoxygenase; SOD, superoxide dismutase; PAO, polyamine oxidase; RBOH, respiratory burst oxidase homologue; CAT, catalase; GPX, glutathione peroxidase; APX, ascorbate peroxidase; GR, glutathione reductase; MDAR, monodehydroascorbate reductase

Normally, the enhancement of respiration leads to the overproduction of reactive oxygen species (ROS) [34]. ROS burst is directly proportional to browning [35]. Thus, we first examined the expression of genes encoding enzymes that participate in browning, including polyphenol oxidases (PPOs, EC: 1.10.3.1), phospholipase D (PLD, EC: 3.1.4.4) and lipoxygenase (LOX, EC: 1.13.11.12). We found that all these genes were either highly expressed on Days 3/6 and then decreased or expressed at low levels at first and then increased to the peak level on Day 12 under control conditions but remained relatively stable in the PSK treatment (Fig. 4a), consistent with the observed phenotypes of PEM browning occurring in the nontreatment control but not in the PSK treatment (Fig. 1a). Then, we further examined the expression of genes functioning in dynamic ROS regulation processes, including ROS production and scavenging. Interestingly, we found that either ROS-producing (Fig. 4b) or ROS-scavenging (Fig. 4c) enzymes showed a similar expression pattern to browning-related enzymes. These results indicated a disequilibrium of redox homeostasis in PEM cultures, more likely indicating a shif to a more oxidizing environment, which might lead to PEM browning in the liquid medium without PSK.

### A potential dual effect of PSK on PRXs

**Fig. 5:**
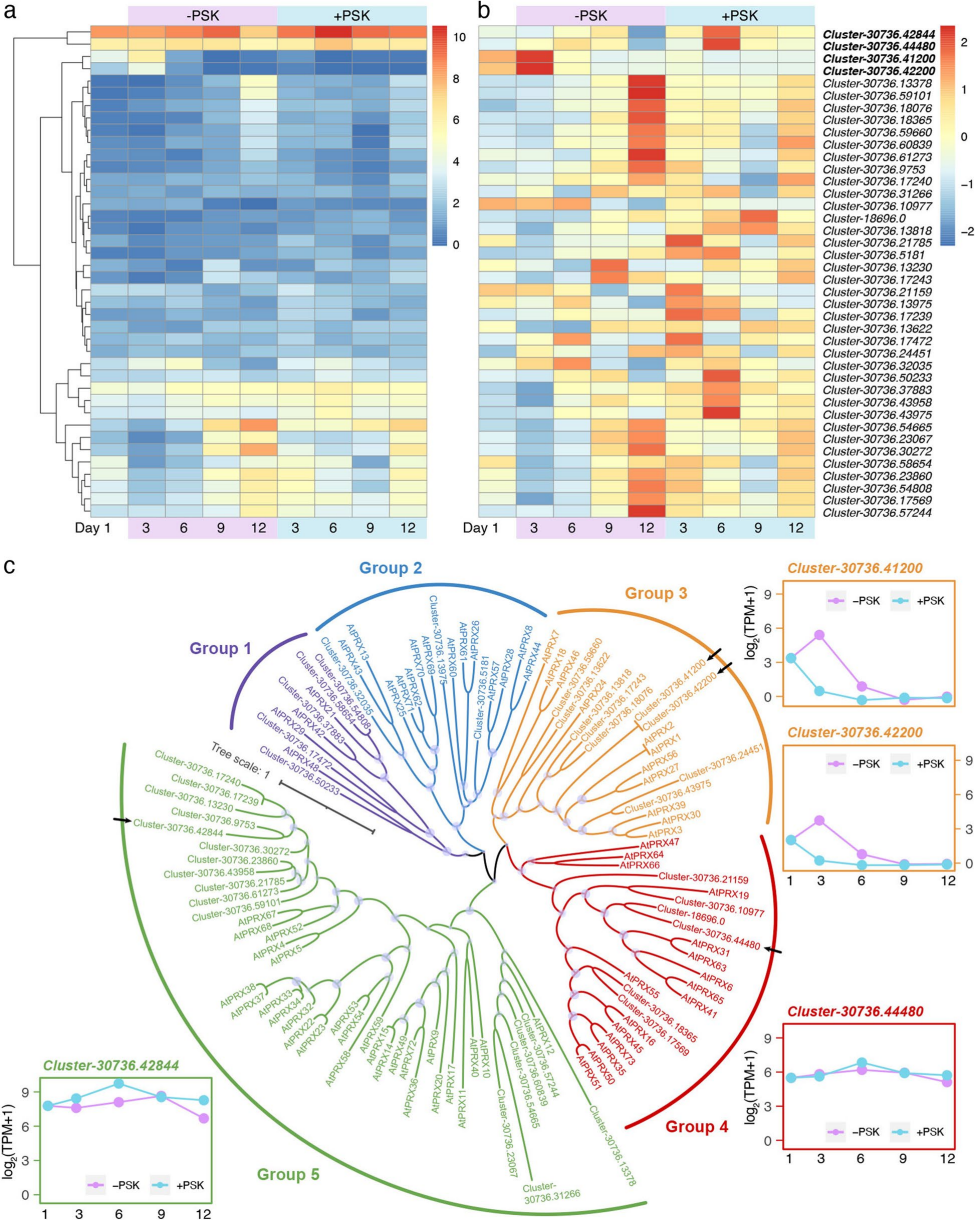
A potential dual effect of PSK on the regulation of ClPRX transcription. Heatmaps of absolute **a** and relative **b** gene expression of *ClPRX*s in *C. lanceolata* PEM cultures with or without PSK treatment. The phylogenetic tree was reconstructed based on the multialignment of ClPRX and AtPRX proteins using the ML method with a bootstrap value of 1,000 **c** Comparisons of gene expression across suspension cultures are shown for four selected unigenes

During expression analyses of redox homeostasis-related genes, we found that Class III peroxidases (PRXs, E1.11.1.7) showed two opposite expression patterns, i.e., one was extremely highly expressed on Days 3/6 or Day 12 in the nontreatment control but maintained stable expression in the PSK treatment, and the other was highly expressed on Days 3/6 in the PSK treatment but expressed at low levels in the nontreatment control (Fig. 5a, b). Since PRXs can act both as ROS scavengers in the peroxidatic cycle and ROS producers in the oxidative and hydroxylic cycles [36], we further examined the phylogenetic classification of these *C. lanceolata PRX* (*ClPRX*) genes by reconstructing an evolutionary tree together with *Arabidopsis* homologues (*AtPRX*s). A total of 72 unigenes were annotated as PRX-coding genes in *C. lanceolata*, of which 64% (46 unigenes) were expressed across the PSK treatment and the nontreatment control. We further predicted protein coding sequences from these expressed PRX-coding unigenes and identified a total of 40 ClPRX preteins by performing a BLASTP search with a best hit to AtPRX in the TAIR database.

Phylogenetic analysis showed that ClPRXs can be classified into five groups together with AtPRXs (Fig. 5c), consistent with the phylogenetic results in *Arabidopsis* [37]. *Cluster-30736.42844* had the overall highest expression level across all these *ClPRX*s (Fig. 5a) and was further transcriptionally activated on Day 6 in the PSK treatment (Fig. 5b). The closest homologue of Cluster-30736.42844 in *Arabidopsis* was AtPRX52 (Fig. 5c), functioning in lignification, which is considered an ROS consumption process via substrate oxidation [38, 39]. Another unigene showing a similar expression pattern to *Cluster-30736.42844* but with a lower absolute expression level was *Cluster-30736.44480*, which was the closest homologue to AtPRX31/63 with no certain biological function. Interestingly, two unigenes (*Cluster-30736.41200* and *Cluster-30736.42200*) shared the same expression pattern, that is, they were specifically transcriptionally activated on Day 3 in the nontreatment control and were clustered together in the phylogenetic tree (Fig. 5). Both of them had the best BLASTP hit to AtPRX3, which was reported to be involved in the production of ROS under potassium deprivation in roots [40]. Overall, these results suggest a potential dual role of PSK in the transcriptional regulation of *PRX*s functioning in ROS production and scavenging, that maintains a redox homeostasis in *C. lanceolata* PEM cultures and prevents PEM browning.

### PSK safeguarded the success of PEM suspension culture

**Fig. 6:**
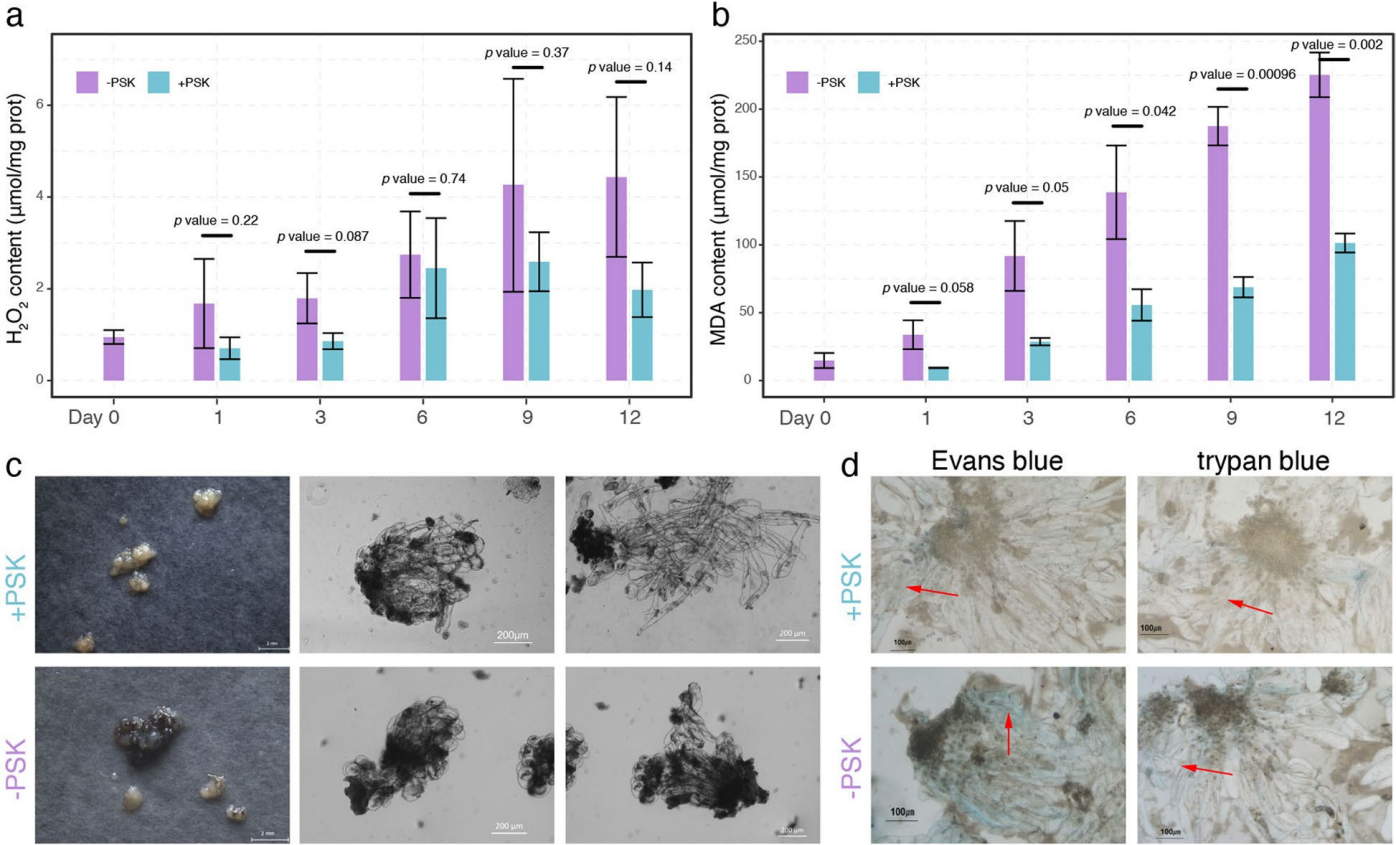
PSK contributes to the establishment of a PEM suspension culture system in *C. lanceolata*. The H_2_O_2_ **a** and MDA **b** contents of PEMs were quantified during suspension culture under both conditions. A t test was performed to examine the difference between the PSK treatment and the nontreatment control at each time point. PEMs were subcultured on solid medium after suspension culture **c** PEMs on solid medium were stained using Evans blue stain and Trypan blue stain **d**

To further confirm the success of *C. lanceolata* PEM suspension culture, we examined the H_2_O_2_ and malondialdehyde (MDA) contents of PEM cultures (Fig. 6a, b). We found that the content of H_2_O_2_ was increased in the suspension culture under both conditions but was always lower in the PSK treatment than in the nontreatment control, although not all comparisons showed a significant difference (Fig. 6a). However, an obvious H_2_O_2_ burst was observed as soon as PEMs were transferred into the liquid medium in the nontreatment control, whereas the H_2_O_2_ content even slightly decreased in the PSK treatment compared with the initial PEMs. In addition, the measurement of MDA content revealed a similar pattern, with much more obvious differences (Fig. 6b). Then, we collected PEM cultures after one cultivation cycle of suspension culture and subcultured them on solid medium. The results showed that PEMs in the PSK treatment continued to grow, whereas browning PEMs tended to die in the nontreatment control (Fig. 6c). Light microscopy showed that a typical PEM structure was maintained in PEMs from PSK treatment, but only broken and incomplete PEMs were found in the nontreatment control (Fig. 6c). Furthermore, Evans blue and Trypan blue staining indicated much more cell viability in PEMs in the PSK treatment than in the nontreatment control (Fig. 6d). Overall, we concluded that PSK helped to establish a PEM suspension culture system in *C. lanceolata* by maintaining ROS homeostasis, inhibiting PEM browning and promoting cell viability.

## Discussion

### PSK inhibits *C. lanceolata* PEM browning during suspension culture

In our research, the major obstacle in the establishment of an effective PEM suspension culture system in recalcitrant *C. lanceolata* genotype 4098–5 was lethal browning after the PEMs were transferred from solid to liquid medium (Fig. 1). Browning requires the joint participation of PPOs and their substrates phenolic compounds, as well as oxygen [17]. Excessive accumulation of ROS induces cell membrane lipid peroxidation and causes structural integrity loss of the cell membrane, leading to cellular decompartmentalization and finally enzymatic browning [35]. Consistent with this, the H_2_O_2_ content increased as soon as PEMs were transferred from solid to liquid medium under control conditions (Figs. 1b and 6a), and the degree of PEM browning increased over the course of the suspension culture (Fig. 1a). In contrast, exogenous application of PSK obviously alleviated the ROS burst, especially in the early stages of the suspension culture (Figs. 1b and 6a), and effectively inhibited PEM browning (Fig. 1a). In particular, PEMs could still grow on the solid medium after a cycle of suspension culture in the PSK treatment, while PEMs in the nontreatment control experienced severe browning and ultimately death (Fig. 6). Thus, exogenous application of PSK effectively inhibits PEM browning and contributes to the establishment of a suspension culture system in this recalcitrant *C. lanceolata* genotype.

### PSK inhibits PEM browning via the maintenance of redox homeostasis

Compared to the nontreatment control, in suspension PEM cultures with additional exogenous PSK, the H_2_O_2_ content was decreased, and browning was inhibited. To explore the underlying transcription regulation mechanisms, we performed comparative time-series transcriptome analyses and found that extensive transcriptional changes occurred as soon as PEMs were transferred from solid to liquid medium under control conditions (Fig. 2). In contrast, exogenous application of PSK facilitated a smooth transition from solid culture to liquid suspension culture at least at the transcriptional level (Fig. 2). GO and KEGG enrichment analyses showed that an enhanced aerobic respiration occurred when PEMs were cultured in liquid suspension, especially at the early stage, in the nontreatment control but not in the PSK treatment (Fig. 3). The enhancement of respiration in the nontreatment control is supposed to induce ROS bursts and browning [34, 35], which is consistent with observations in the practical situations of our study (Fig. 1). Furthermore, we examined the transcriptional responses of key enzymes in ROS production and scavenging to suspension culture and found that most of these genes presented dramatic fluctuations in the nontreatment control but maintained a relatively stable expression pattern in the PSK treatment (Fig. 4).

Recent studies have shown that the PSK receptor RSKR1 can enhance plant defence and growth via phosphorylation of a glutamine synthetase in tomato, which is associated with oxidation–reduction processes [28]. In peach fruit, exogenous application of PSK contributed to a decrease in ROS content and LOX activity, thus ameliorating chilling injury [30]. Furthermore, exogenous PSK in banana helps to alleviate chilling injury by increasing polyamine, proline, and γ-aminobutyric acid (GABA), all of which play a role in mitigating ROS in plants [29, 41–43]. In addition, exogenous PSK application decreased H_2_O_2_ and MDA accumulation, accompanied by higher expression of *SOD*, *CAT*, *APX*, and *GR* and lower expression of *PLD* and *LOX*, thus delaying broccoli floret yellowing during cold storage [44]. Consistent with the roles of PSK in the regulation of redox homeostasis, we found that PSK decreased the H_2_O_2_ and MDA content and inhibited browning in PEM cultures during suspension culture. However, exogenous PSK contributed to redox homeostasis via the maintenance of the stability of ROS metabolism, rather than some particular ROS-producing or ROS-scavenging enzymes in *C. lanceolata* PEM culutres (Fig. 4). In other words, PSK treatment decreased the transcriptional responses of most ROS-producing and ROS-scavenging enzymes to the transition from solid to liquid medium, maintaining a relatively stable redox environment.

## Conclusions

In summary, we established a PEM suspension culture system in a recalcitrant *C. lanceolata* genotype, in which H_2_O_2_ and MDA accumulation and successive PEM browning were repressed by exogenous application of PSK. We performed comparative time-series transcriptome analysis and found that exogenous PSK contributed to the smooth transition of PEM cultures from solid to liquid medium at least at the transcriptional level. Further gene expression profiling showed that PSK treatment decreased the transcriptional fluctuations of most ROS metabolism-related enzymes, thus maintaining redox homeostasis when PEMs were cultured in liquid suspension, especially in the early stage. In addition, we also found that PSK might have a dual effect on the transcriptional regulation of *PRX*s, i.e., repressing ROS-producing *PRX*s and activating ROS-scavenging *PRX*s. These results provide new insights into the biological roles of PSK in repressing browning during plant cell suspension culture and a valuable tool for future establishment of a large-scale suspension culture system of conifer species without genotype limitation.

## Methods

### Plant materials

Immature cones were collected from a clonal tree of *C. lanceolata* genotype 4098 growing at the Yangkou forest station of the Chinese fir National Germplasm Bank (Fujian, China) in 2017. Renhua Zheng and Jisen Shi were responsible for the formal identification of the samples. Living plantlets are preserve in the Chinese fir National Germplasm Bank (Fujian, China) with a genotype number of ‘4098’. PEMs were induced from these cones and subcultured in the State Key Laboratory of Tree Genetics and Breeding (Nanjing, China). In this study, approximately 2 g of PEMs on the 21st day of growth on solid medium was subcultured in liquid medium at an agitation speed of 85 rpm for suspension culture in 50 mL Erlenmeyer flasks. The liquid suspension medium consisted of DCR salts, 0.5 mg/L 2,4-D, 0.2 mg/L 6-BA, 10 mg/L VC, 450 g/L glutamine, 500 mg/L CH, 2 g/L inosite and 30 g/L maltose. The treatment concentration of PSK was 0.2 mg/L. RNA samples from mixed PEM cultures at 1, 3, 6, 9, and 12 days in liquid medium with and without of PSK were prepared for RNA-seq with three biological replicates per sample.

### Transcriptome analysis

The quantity and quality of RNA was assessed using the RNA Nano 6000 Assay Kit of the Bioanalyzer 2100 system (Agilent Technologies, USA), and approximately 1.5 μg RNA per sample was used as the input material for the RNA sample preparations. Sequencing libraries were generated and sequenced by Illumina NovaSeq 6000, generating the end reading of 150 bp pairing. After quality control, read filtering and base correction for the raw read data, the clean read data were used to de novo assemble transcripts using Trinity version 2.6.6 [45]. Then, Corset version 4.6 was used to aggregate redundant transcripts, which were used as representative gene models in the following differential expression analysis [46]. We used the clean read data to quantify representative gene model expression using Kallisto version 0.46.1 [47]. Then, we used the R package edgeR version 3.34.1 to perform differential expression analysis using read count data [48]. Specifically, we performed one-way analysis of variance (ANOVA)-like testing using the glmQLFTest function in edgeR to identify differentially expressed genes across all samples with an FDR cut-off of 0.05. Next, we used the R package clusterProfiler version 4.0.5 to perform GO and KEGG enrichment analyses [49] and pheatmap version 1.0.12 to draw gene expression heatmaps.

### *PRX* gene family identification

All CDS and protein sequences of Class III PRXs in *Arabidopsis* were downloaded from The Arabidopsis Information Resource (TAIR, www.arabidopsis.org) [50]. Then, these *AtPRX* nucleic acid sequences were used in a BLASTN search against the *C. lanceolata* redundant transcript database, resulting in *ClPRX* candidates with E-values less than 0.0001 [51]. After that, each *ClPRX* candidate was submitted to NCBI ORFfinder (https://www.ncbi.nlm.nih.gov/orffinder/) for predicting open reading frames (ORFs) and to NCBI CDD version 3.20 for predicting conserved domain [52]. The predicted protein sequence was further submitted to TAIR to run a BLASTP search and was counted as a ClPRX protein if the best BLASTP hit was an AtPRX protein. Finally, all PRX protein sequences were aligned using Clustal Omega version 1.2.4 [53]. The phylogenetic tree was reconstructed using RAxML version 8.2.11 and visualized using iTOL version 6.7.4 [54, 55].

### ROS staining and measurement

Histochemical staining of H_2_O_2_ was performed using DAB solution (Sigma‒Aldrich) at a working concentration of 1 mg/ml. *C. lanceolata* PEMs were infiltrated with DAB solution and incubated in the dark for 1.5 h at room temperature. The stained PEMs were removed and made into slides, which were observed and photographed using an inverted microscope (Leica, DMI4000) and a confocal microscope (Carl Zeiss, LSM 800). The H_2_O_2_ content was determined using an H_2_O_2_ assay kit (Jiancheng, China), and the absorbance was measured at 415 nm using an enzyme labelling instrument with three biological replicates. The MDA content was determined using an MDA assay kit (Jiancheng, China), and the absorbance was measured at 532 nm using an enzyme labelling instrument with three biological replicates.

### Evans blue and trypan blue staining

*C. lanceolata* PEMs were extracted from liquid suspension medium and placed on slides, stained with Evans blue stain (0.5%) or Trypan blue stain (0.04%) for 45 s, and observed and photographed using a confocal microscope (Carl Zeiss, LSM 800).

### Supplementary Information

**Additional file 1.** **Table S1.** Data statistics of RNA-seq data. **Table S2.** Length distribution of assembled transcripts and unigenes. **Table S3.** BUSCO assessment for transcript assembly completeness.

## Data Availability

Raw reads have been deposited under National Center for Biotechnology Information (NCBI) BioProject accession number PRJNA970264.
